# Signalment and C-reactive protein values in dogs with immune-mediated polyarthritis and steroid responsive meningitis arteritis

**DOI:** 10.3389/fvets.2023.1091318

**Published:** 2023-02-14

**Authors:** Viktoriya Indzhova, Michał Czopowicz, Scott Kilpatrick, Rodrigo Gutierrez-Quintana, Josep Brocal

**Affiliations:** ^1^Willows Veterinary Centre and Referral Service, Part of Linnaeus Veterinary Limited, Solihull, United Kingdom; ^2^Division of Veterinary Epidemiology and Economics, Institute of Veterinary Medicine, Warsaw University of Life Sciences—SGGW, Warsaw, Poland; ^3^The Veterinary Thought Exchange, Galston, United Kingdom; ^4^School of Veterinary Medicine, College of Medical, Veterinary and Life Sciences, University of Glasgow, Glasgow, United Kingdom; ^5^Anderson Moores Veterinary Specialists, Part of Linnaeus Veterinary Limited, Winchester, United Kingdom

**Keywords:** canine (dog), inflammation, acute phase protein (APP), SRMA (steroid-responsive meningitis arteritis), IMPA, C-reactive protein

## Abstract

**Introduction:**

This retrospective multicentric study aims to evaluate the ability of CRP concentration to differentiate between dogs diagnosed with IMPA and SRMA. C-reactive protein (CRP) is a marker of inflammation widely used in two of the most commonly diagnosed immune-mediated diseases in dogs—Immune-mediated polyarthritis (IMPA) and steroid responsive meningitis arteritis (SRMA).

**Materials and methods:**

Data collected from medical records of 167 client-owned dogs included age, breed, gender, neuter status, body weight, body temperature, CRP concentration, month and season of diagnosis. CRP was measured quantitatively in 142 dogs (84%) and semi-quantitatively in 27 dogs (16%).

**Results:**

SRMA was diagnosed significantly more often in dogs < 12 months old and IMPA in dogs ≥12 months old (*P* < 0.001). Dogs diagnosed with SRMA had higher CRP concentration than dogs diagnosed with IMPA (*P* = 0.02). This difference was influenced by the dog's age–when a dog was <12 months old, a higher CRP concentration indicated IMPA (*P* = 0.02), whereas when a dog was ≥12 months old, a higher CRP concentration indicated SRMA (*P* = 0.02).

**Discussion:**

CRP concentration as a sole diagnostic modality showed only fair discriminatory potential to differentiate between SRMA and IMPA (area under ROC curve close to 0.7). CRP concentration varied depending on patient age and definitive diagnosis. It may play some role in differentiating between SRMA and IMPA but should not be used as the sole diagnostic modality, given it has been demonstrated to only have fair discriminatory potential.

## Introduction

Immune-mediated polyarthritis (IMPA) and steroid responsive meningitis arteritis (SRMA) are two of the most commonly diagnosed immune-mediated diseases in dogs (1). SRMA typically affects dogs younger than 2 years of age, with a median age of 10 months (2, 3), while IMPA typically affects older dogs with a median age of 4 years (2, 4). For both diseases, certain breeds are overrepresented: Beagles, Bernese Mountain Dogs, Border Collies, Boxers, English Springer Spaniels, Jack Russell Terriers, Nova Scotia Duck Tolling Retrievers, Weimaraners, Whippets for SRMA (2–6) and Rottweilers, Labrador Retrievers, Golden Retrievers, Shetland Sheepdogs, Irish Setters, Cocker Spaniels, and American Eskimo for IMPA (7–9). The source of pain may not always be obvious in both conditions; dogs with SRMA typically present with neck pain, whereas dogs suffering from IMPA typically present with appendicular joint pain. However, dogs with IMPA may also have cervical and thoracolumbar spinal pain, therefore distinguishing between both conditions based on physical examination alone can be challenging (1). Furthermore, a subset of dogs will suffer from both conditions concomitantly (1). Pyrexia is typically identified in patients with either condition. It is therefore of clinical relevance to distinguish between both conditions as this will subsequently guide investigations and therefore influence treatment. Although a tapering course of prednisolone is used to treat both conditions, the choice of second immune-suppressive medication may vary depending on the disease process (2, 10), further highlighting the importance of distinguishing between SRMA and IMPA.

The exact pathogenesis of SRMA and IMPA remains unclear, but both are immune-mediated diseases. IMPA is believed to be a type III hypersensitivity reaction where immune complexes accumulate in the joint space leading to activation of the complement, release of cytokines and attraction of neutrophils and lymphocytes causing tissue damage (11). SRMA is characterized by neutrophilic pleocytosis and an arteritis particularly in the cervical leptomeninges. Cytokines and integrin and matrix metalloproteinase upregulation aid in causing blood-brain-barrier disruption and leucocyte migration into the subarachnoid space causing fibrinoid arteritis and leptomeningeal inflammation. Transforming growth factor beta 1 (TGF-β_1_), interleukin-6 (IL-6), CD11a, vascular endothelial growth factor (VEGF), IgA, matrix metalloproteinase-2 (MMP-2) and Th17 have all been described to play a role in the pathology of leading to vascular wall destruction, development of fever, activation of acute phase proteins, neutrophilic pleocytosis and leucocytosis (12–18).

Injury to the tissues results in a response that includes changes in the concentration of certain plasma proteins called acute phase proteins (APPs) (19). C-reactive protein (CRP) is a major APP in dogs (20). CRP is a non-specific APP, but can be markedly elevated in infectious and immune-mediated inflammatory diseases (21, 22). Serum CRP has been shown to be significantly increased at the time of diagnosis of SRMA and IMPA, with subsequent decreasing concentration after successful treatment and resolution of the clinical signs (2, 4). Serum CRP has also been shown to have a significant positive correlation with the total nucleated cell count of the cerebrospinal fluid (CSF) in dogs diagnosed with SRMA, and a modest positive correlation with joint cellularity in dogs diagnosed with IMPA (2, 4). No significant age- and sex-related differences are found in the CRP values of healthy dogs (23, 24), but healthy Miniature Schnauzer dogs have slightly higher CRP concentration in comparison to other dog breeds (25). Correlations of CRP values from dogs suffering from SRMA or IMPA with age, gender, neutered status, body weight, month and season at the time of diagnosis and differences between breeds have not been explored. Furthermore, CRP values in dogs diagnosed with SRMA and IMPA have never been directly compared on large groups of dogs, probably because results from different studies with low numbers suggest similar results (2, 4).

The aim of this study was to determine the role of CRP values in distinguishing between dogs diagnosed with SRMA and IMPA.

## Materials and methods

A retrospective review of the medical records of dogs diagnosed with non-infectious, non-erosive, idiopathic IMPA and SRMA from two referral institutions during the period between 2017 and 2021 was performed. The terms used as identifiers during the search were: “immune-mediated polyarthritis,” “steroid-responsive meningitis arteritis,” “meningitis,” “arthritis,” “polyarthritis,” “SRMA,” “IMPA.” Patients were included if they were diagnosed with non-infectious, non-erosive, idiopathic (primary) IMPA or SRMA and CRP was measured at the time of presentation. Ethical approval was granted by the Research Ethics Committee at the University of Glasgow with a reference number EA39/20.

A diagnosis was made based on consistent medical history, physical, neurological and orthopedic examination, and clinicopathologic findings (results of hematology, serum biochemical analysis for IMPA and SRMA, and thoracic and abdominal imaging for IMPA). These were coupled with the results of routine analysis of CSF collected from the cerebellomedullary or lumbar cistern. For those patients identified for the purposes of the study with SRMA, fluid analysis revealed neutrophilic or mixed neutrophilic and monocytic pleocytosis with no visible organisms, and lack of toxic neutrophils (1, 2). Patients diagnosed with IMPA were identified for the purposes of the study as those whose results of synovial fluid analysis indicated neutrophilic inflammation in two or more joints.

Other diagnostic tests performed in an attempt to rule out other causes of CSF pleocytosis (imaging of the vertebral column, PCR assays to detect *Toxoplasma gondii, Neospora caninum*, canine distemper virus in CSF, CSF culture and serum *T. gondii and N. caninum* antibody titers) or secondary IMPA (echocardiography, serologic and PCR assays to detect vector-borne disease, serologic testing for *Bartonella* sp. infection, joint radiography, and microbial culture of blood, synovial fluid, or urine samples) were performed at the discretion of the attending clinician, taking into consideration patient demographic and historical factors, physical examination findings, preliminary test results, and client finances. Dogs were excluded if they had incomplete medical history, no definitive diagnosis or had undergone corticosteroid treatment prior to diagnosis. Additionally, dogs with SRMA were excluded if they had neurological deficits.

Data retrieved from the medical records was as follows: breed, age, body weight, gender, neutered status, month of presentation, history, physical and neurologic examination findings, CRP values at the time of diagnosis, CSF routine analysis and joint cytology results, imagining modalities performed and results, infectious disease testing, and final diagnosis.

CRP was measured quantitatively in 142 dogs (84%) and semi-quantitatively in 27 dogs (16%). For the purposes of statistical analysis semi-quantitative results were replaced as follows: 2.5 mg/L instead of <5, 150 mg/L instead of >100 mg/L, and 250 mg/L instead of >200 mg/L. The CRP serum concentration was measured using species-specific immunoturbidimetric assay for canine CRP (Gentian Canine CRP Immunoassay, Gentian AS, Moss, Norway) in one of the hospitals and using IDEXX Catalyst^®^ CRP Test (a sandwich immunoassay, IDEXX, USA) in the other. The two assays were compared and considered to provide accurate and consistent results (26). The reference range for CRP was <10 mg/L.

### Statistical analysis

Normality of distribution of numerical variable was investigated visually on histograms and using the Shapiro-Wilk test. As their distribution was markedly right-hand skewed, numerical variables were presented as the median, interquartile range (IQR) and range or individual dog measurements on the plot. The magnitude of difference of a numerical variable between two groups was reported as the median difference with 95% confidence interval (CI 95%) (27). Categorical variables were described as counts and percentages. CI 95% for proportions were calculated using the Wilson score method and the magnitude of difference of a categorical variable between two groups was reported as the difference between proportions with CI 95% (27). The relationships between demographic and clinical variables and diagnosis of SRMA or IMPA, in addition to those relationships between demographic and clinical variables and CRP concentration, were evaluated first in the univariable analysis. The groups were compared using the Mann-Whitney U test (2 groups) or Kruskal-Wallis H test (>2 groups; followed by Dunn's *post-hoc* test if applicable) for numerical variables, and using the maximum likelihood G test or Fisher's exact test (if the expected count in any cell of the contingency table was <5) for categorical variables. Correlations between two numerical or ordinal variables were determined using the Spearman's rank correlation coefficient (*R*_*s*_) with CI 95% (27). Strength of correlation was classed as follows (28): *R*_*s*_ = 0.00 to 0.19-very weak, 0.20 to 0.49-weak, 0.50 to 0.69-moderate, 0.70 to 0.89-strong, and 0.90 to 1.00-very strong. Subsequently, variables significantly (α = 0.05) linked to the diagnosis and CRP concentration were included in the multivariable logistic regression model with backward stepwise elimination procedure evaluating the relationship between CRP and the diagnosis. The strength of relationship was expressed as crude odds ratios (OR) in the univariable analysis and adjusted OR (OR_adj_) in the multivariable analysis. The area under receiver operating characteristic curve (AUROC) was calculated to determine the discriminative potential of CRP concentration and interpreted as poor when <0.7, fair when 0.7–0.8, good when 0.8–0.9, and excellent when >0.9 (29). The optimal cut-off value was based on the highest value of the Youden's index (J) calculated as diagnostic sensitivity (Se) + diagnostic specificity (Sp)−1 (30). A significance level (α) was set at 0.05. In the case of multiple comparisons between subgroups the Bonferroni correction was used to control for the inflation of type I error and the *P*-value in such analyses was denoted as *P*_BC_. All statistical tests were two-tailed. The statistical analysis was performed in TIBCO Statistica 13.3 (TIBCO Software Inc., Palo Alto, CA) and IBM SPSS Statistics 26 (IBM Corporation, Armonk, NY).

## Results

One hundred and sixty-nine dogs were enrolled in the study-−91 males (46 [51%] neutered) and 78 females (39 [50%] neutered). Their ages ranged from 2 months to 15 years, with a median (IQR) of 18 (10–66) months. Males were demonstrated to be significantly younger than females−15 (9–45) months vs. 26 (11–83) months (*P* = 0.02). Entire dogs were demonstrated to be significantly younger than neutered dogs–a median (IQR) of 11 (8–23) months vs. 42 (16–87) months (*P* < 0.001). From among dogs aged <12 months-21.8% were neutered (12/55), 12–24 months-50% (22/44) were neutered, and >24 months-73% (51/70) were neutered. Eighty-five dogs had SRMA and 84 had IMPA. Demographic and clinical characteristics of dogs with the two diagnoses are presented in Table 1. Briefly, dogs with SRMA were significantly younger than dogs with IMPA (*P* < 0.001)−95% of dogs with SRMA were ≤24 months old while 79% of dogs with IMPA were >24 months old. As a result, a significantly higher proportion of dogs with IMPA were neutered (*P* = 0.003). Pedigree dogs accounted for majority of dogs in both groups. The most common breeds with SRMA were Cockapoo, Whippet, and Beagle, while Cocker and Springer Spaniel with IMPA. Twenty-eight different dog breeds were affected by SRMA and of 42 different breeds were affected by IMPA (Supplementary Table S1). Regardless of the diagnosis, most dogs (70–80%) were presented with pyrexia, rarely >40.5°C. Both diseases were diagnosed with uniform frequency throughout the four seasons.

**Table 1:** Univariable analysis of the relationship between demographic and clinical variables and the diagnosis of steroid responsive meningitis-arteritis (SRMA) and immune-mediated polyarthritis (IMPA).

**Variable^a^**	**SRMA (*n =* 85)**	**IMPA (*n =* 84)**	***P*-value**
**Demographic characteristics**			
Age [month]	11, 8–16 (4–68)	65, 32–95 (2–182)	<0.001^*^
**Age class**			<0.001^*^
<12 months	45 (53)	10 (12)	
12–24 months	36 (42)	8 (9)	
>24 months	4 (5)	66 (79)	
**Sex**			0.32
Males	49 (58)	42 (50)	
Females	36 (42)	42 (50)	
**Neuter status**			0.003^*^
Entire	52 (61)	32 (38)	
Neutered	33 (39)	52 (62)	
**Breed**			
Cross-breed	12 (14)	8 (9)	-
Pedigree	73 (86)	76 (91)	0.35
Cockapoo	12 (14)	3 (4)	0.20
Whippet	9 (11)	2 (2)	0.26
Beagle	9 (11)	1 (1)	0.20
Cocker spaniel	3 (4)	8 (9)	0.08
Springer spaniel	2 (2)	7 (8)	0.11
Body weight [kg]	13.5, 9.3–39.6 (3.1–45.4)	15.5, 8.5–26.3 (2.5–63.4)	0.07
**Clinical characteristics**			
Body temperature [°C]	39.7, 39.3–40.0 (37.5–41.1)	39.6, 39.0–40.0 (37.5–41.2)	0.60
**Body temperature class**			0.40
Normal (37.5–39.2°C)	18 (22)	20 (28)	
Moderate pyrexia (39.2–40.4°C)	58 (71)	43 (61)	
Marked pyrexia (≥40.5°C)	6 (7)	8 (11)	
**Season when diagnosed**			
Spring	23 (27)	16 (19)	0.42
Summer	20 (24)	28 (33)	0.11
Autumn	19 (22)	20 (24)	0.36
Winter	23 (27)	20 (24)	0.62

^*^Significant at α = 0.05.

^a^Numerical variables presented as median, IQR (range), and categorical variables as counts and percentages.

CRP concentration ranged from 1 to 1,398 mg/L with the median (IQR) of 141 (85–175) mg/L and it was elevated > 10 mg/L in 97% of dogs (163/169). CRP concentration was significantly negatively correlated with age, however this correlation was only weak (*R*_*s*_= −0.19; CI 95%: −0.04 to −0.33): there was no significant difference in CRP concentration between dogs <12 months and 12–24 months (median difference = −11 mg/L; CI 95%: −49 to 9 mg/L; *P* = 0.99), while CRP concentration in dogs >24 months was significantly lower than in the two former groups (median difference = −40 mg/L; CI 95%: −60 to −10 mg/L; *P* = 0.003). Given that proportion of neutered dogs increased with age, CRP concentration was significantly higher in entire than in intact dogs (median difference = 24 mg/L; CI 95%: 0 to 50 mg/L; *P* = 0.04).

CRP concentration was significantly positively correlated with body temperature (*R*_*s*_ = 0.36; CI 95%: 0.22 to 0.49; *P* < 0.001), both in dogs with SRMA (*R*_*s*_ = 0.36; CI 95%: 0.15 to 0.53; *P* = 0.001) and IMPA (*R*_*s*_ = 0.37; CI 95%: 0.15 to 0.55; *P* = 0.002). However, there was no significant difference in CRP concentration between dogs with moderate (39.2 to 40.4°C) and marked pyrexia (≥40.5°C) (median difference = 0 mg/L; CI 95%: −44 to 41 mg/L; *P* = 0.99) (Table 2).

**Table 2:** Univariable analysis of the relationship between demographic and clinical variables and the CRP concentration [mg/L].

**Variable**	**CRP concentration [mg/L]^a^**	***P*-value**
**Demographic characteristics**		
Age [mth]	R_s_ = −0.19 (CI 95%: −0.04 to −0.33)	0.01^*^
**Age class**		0.01^*^
<12 months (*n =* 55)	146, 96.3–174 (2.5–456)	
12–24 months (*n =* 44)	150, 93.7–201 (12.9–471)	
>24 months (*n =* 70)	95.7, 56.1–150 (1.0–1,398)	
**Sex**		0.18
Females (*n =* 78)	110, 59.4–156 (1.0–1,398)	
Males (*n =* 91)	146, 86.4–193 (9.3–426)	
Neuter status		0.04^*^
Entire (*n =* 84)	150, 95.4–175 (2.5–471)	
Neutered (*n =* 85)	100, 58.3–170 (1.0–1,398)	
**Breed**		0.31
Cross-breed (*n =* 20)	145, 93.6–197 (10.2–1,398)	
Pedigree (*n =* 149)	138, 81.5–170 (1.0–471)	
Cockapoo (*n =* 15)	144, 50.0–168 (2.5–250)	0.55
Whippet (*n =* 11)	141, 49.0–150 (21.7–174)	
Beagle (*n =* 10)	90.0, 13.8–188 (9.3–426)	
Cocker spaniel (*n =* 11)	150, 143–191 (96.8–225)	
Springer spaniel (*n =* 9)	133, 91.0–150 (81.5–328)	
**Clinical characteristics**		
Body weight [kg]	R_s_ = 0.09 (CI 95%: −0.06 to 0.24)	0.25
Body temperature [°C]	R_s_ = 0.36 (CI 95%: 0.22 to 0.49)	<0.001^*^
**Fever**		0.001^*^
Normal (37.5–39.2°C) (*n =* 38)	95.7, 52.1–144 (9.1–328)	
Moderate fever (39.2–40.4°C) (*n =* 101)	150, 92.2–201 (2.5–471)	
High fever (≥40.5°C) (*n =* 14)	150, 138–174 (56.4–1,398)	
**Season**		0.49
Spring (*n =* 39)	150, 91.6–183 (1.0–456)	
Summer (*n =* 48)	10.4, 58.5–157 (8.1–1,398)	
Autumn (*n =* 39)	140, 84.8–199 (9.3–365)	
Winter (*n =* 43)	146, 81.5–175 (1.5–471)	

^*^Significant at α = 0.05.

^a^CRP presented as median, IQR (range).

**Figure 1:**
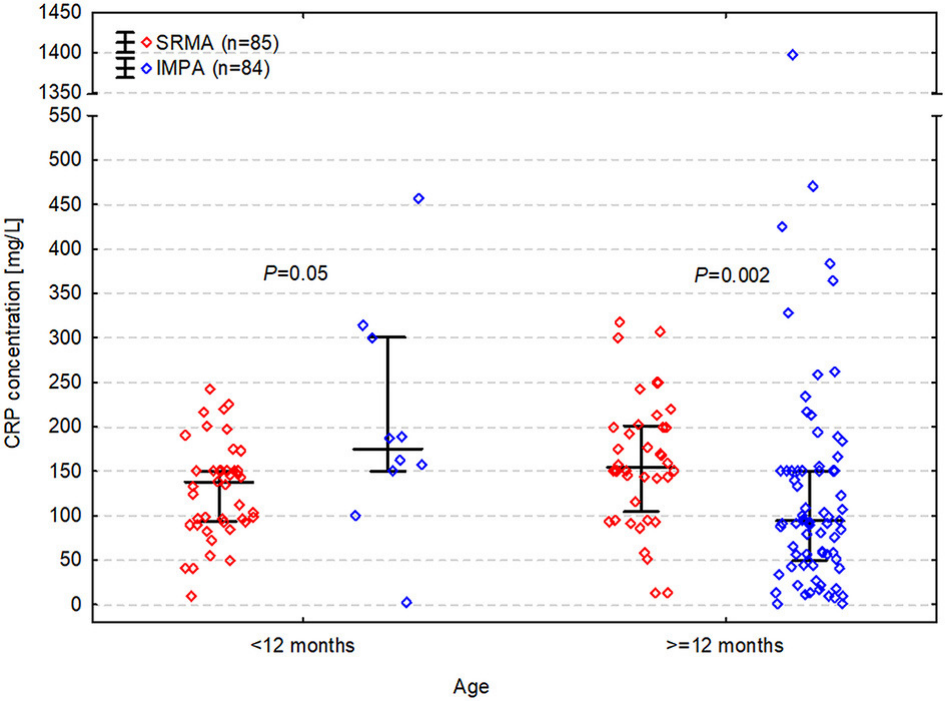
C-reactive protein (CRP) concentration in dogs with steroid responsive meningitis-arteritis (SRMA) and immune-mediated polyarthritis (IMPA) depending on age.

CRP concentration was significantly higher in dogs with SRMA (median [IQR] of 150 [95–176] mg/L) than in dogs with IMPA (median [IQR] of 99 [54–160] mg/L) and the median difference was 35 mg/L (CI 95%: 3 to 55 mg/L; *P* = 0.02). It was elevated >10 mg/L in all dogs with SRMA (100%; CI 95%: 96 to 100%) and in 93% (CI 95%: 85 to 96%) of dogs with IMPA (78/84). The difference between percentages was 7% (CI 95%: 1 to 15%) and was significant (*P* = 0.01). Nevertheless, the relationship between CRP concentration and the diagnosis appeared to depend on dog's age. CRP concentration was significantly higher in SRMA than in IMPA in dogs ≥12 months (median difference = 53 mg/L; CI 95%: 18 to 85 mg/L; *P*_BC_ = 0.002), whereas it was significantly lower in SRMA than in IMPA in dogs <12 months (median difference = −58 mg/L; CI 95%: −8 to −133 mg/L; *P*_BC_ = 0.05) (Figure 1). Therefore, in the multivariable logistic regression evaluating the relationship between CRP concentration and diagnosis along with age and neuter status, an interaction between CRP concentration and age (categorized as <12 months and ≥12 months) was also included.

Two variables proved significant in the multivariable analysis (Table 3): Age in dogs ≥12 months IMPA was significantly more likely than SRMA (OR_adj_ = 0.014; CI 95%: 0.002 to 0.105; *P* < 0.001); and CRP concentration which was, however, modified by age–when a dog was <12 months old, a higher CRP concentration indicated IMPA (OR_adj_ = 0.988; CI 95%: 0.977 to 0.998; *P* = 0.02), whereas when a dog was ≥12 months old, a higher CRP concentration indicated SRMA (OR_adj_ = 1.014; CI 95%: 1.003 to 1.025; *P* = 0.02). This multivariable model fit the data well (Nagelkerke's pseudo-R^2^ coefficient = 0.29) and had fair discriminatory ability (AUROC of 80%; CI 95%: 74% to 87%).

**Table 3:** Multivariable logistic analysis of the relationship between C-reactive protein (CRP) and diagnosis of steroid responsive meningitis arteritis (SRMA) (an effect category in the logistic regression) and immune-mediated polyarthritis (IMPA) (a reference category in the logistic regression) controlled for potential confounders.

**Variable**	**Regression coefficient (standard error)**	**Wald's statistic**	***P*-value**	**Adjusted odds ratio (95% confidence interval)**
Intercept	3.48 (0.99)	-	-	-
Age ≥ 12 months	−4.27 (1.03)	17.2	<0.001*	0.014 (0.002 to 0.105)
CRP concentration	−0.012 (0.005)	5.15	0.02*	0.988 (0.977 to 0.998)
Interaction between CRP and Age ≥ 12 months	0.014 (0.006)	5.82	0.02*	1.014 (1.003 to 1.025)
Variables dropped as insignificant:				
Neutered	−0.43 (0.37)	1.34	0.25	0.65 (0.31 to 1.35)

^*^Significant at α = 0.05

**Figure 2:**
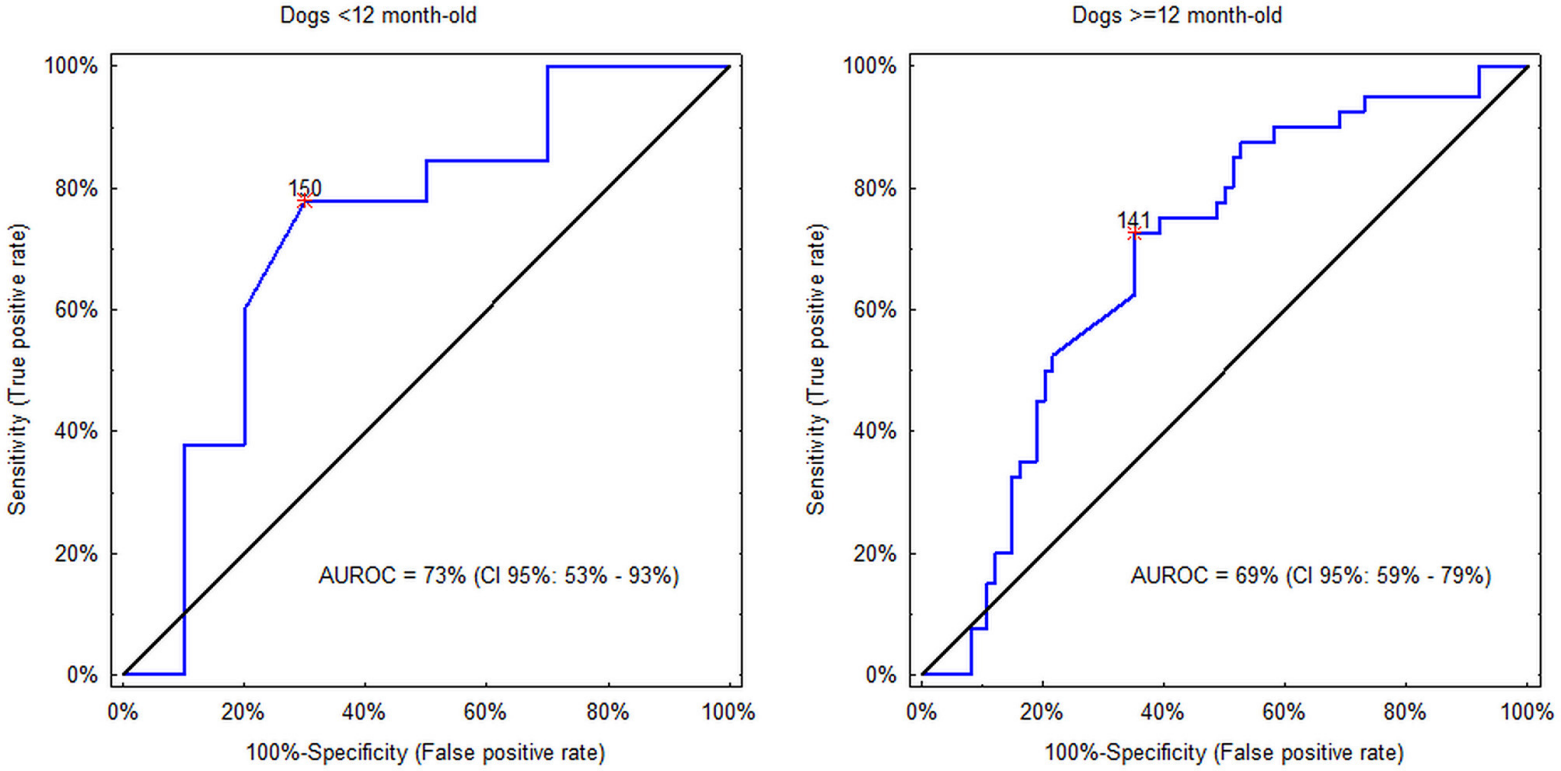
Discriminatory potential of C-reactive protein (CRP) concentration between steroid responsive meningitis-arteritis (SRMA) and immune-mediated polyarthritis (IMPA) in dogs <12 months and ≥12 months. Asterisks indicate the optimal cut-off values. In the former group values >150 mg/L indicate IMPA, whereas in the latter group values >140 mg/L indicate SRMA.

Although CRP proved to be independently linked to the diagnosis, in each of the two age classes (<12 months vs. ≥12 months) CRP concentration as a sole diagnostic modality showed only fair discriminatory potential between SRMA and IMPA with AUROCs around 0.7, and the cut-off equal to 140–150 mg/L (Figure 2). In dogs <12 months CRP concentration >150 mg/L was detected in 22% (CI 95%: 13% to 36%; 10/45) of dogs with SRMA and in 70% (CI 95%: 40% to 89%; 7/10) of dogs with IMPA (difference between proportions = −48%; CI 95%: −14% to −69%; *P*_BC_ = 0.01). CRP concentration > 150 mg/L was associated with roughly 8-fold increase of odds of IMPA (OR = 8.2; CI 95%: 1.8 to 37.5). On the other hand, in dogs ≥12 months CRP concentration >140 mg/L was detected in 73% (CI 95%: 57% to 84%; 29/40) of dogs with SRMA and in 37% (CI 95%: 26% to 48%; 27/74) of dogs with IMPA (difference between proportions = 36%; CI 95%: 17% to 51%; *P*_BC_ <0.001). CRP concentration > 140 mg/L was associated with approximately 5-fold increase of odds of SRMA (OR = 4.6; CI 95%: 2.0 to 10.6).

## Discussion

This study describes, for the first-time, CRP concentration in a large cohort of dogs diagnosed with SRMA and IMPA. Furthermore, the study also explored possible relationships between these diseases and CRP concentration with demographic and environmental variables. Dogs diagnosed with SRMA had a significantly higher CRP concentration than dogs diagnosed with IMPA. However, the character of this difference used to change along with dog's age (i.e., age appears to influence the relationship between CRP concentration and the two diseases). In young dogs (<12 months) higher CRP indicated IMPA, whereas in older dogs higher CRP indicated SRMA. In dogs <12 months, IMPA is less likely than SRMA. This is in contrast to a population of older dogs in which IMPA is a much more likely diagnosis. Therefore, measuring CRP may play some role in facilitating the practical diagnosis of these two conditions. The elevation (approximately above 140–150 mg/L) may be used as an indicator of this diagnosis which occurs less often in a given age class. CRP concentrations should, however, not be used as a sole diagnostic modality to differentiate between SRMA and IMPA, given the strength of its discriminatory potential is only fair (29).

Although, the typical age of dogs affected with SRMA and IMPA is different, dogs of any age can be affected by both diseases and clinical signs can be very similar (1). Therefore, the aim of the study was to explore whether CRP could be used as a marker to distinguish between most cases of SRMA and IMPA.

Age can have a significant impact on the immune response (31). It has been demonstrated that *ex-vivo* whole blood samples taken from geriatric dogs have a reduced increase in some inflammatory mediators compared to the blood from middle-aged dogs (31). Autoimmune diseases in elderly people are also often rarer and milder compared to the young population (32). APPs such as CRP are synthesized by the hepatocytes under the influence of interleukin-6, interleukin-1 and tumor necrotizing factor alfa (21). However, most studies in healthy and diseased dogs showed that the CRP concentration and the activation of inflammatory mediators are closely linked (20). However, most studies in healthy and diseased dogs showed CRP values were not affected by age or sex (3, 20, 24). These studies included mostly adult dogs, and studies making comparisons with puppies are scarce; however, these studies which enrolled also dogs <12 months of age suggest that changes of CRP concentration may differ between young and older dogs (33). Previous studies have not found differences in the CRP concentration of healthy Beagles and dogs with various diseases between sexes. Although we identified a difference between neutered status and diagnosis, this relationship was likely to be associated with the fact that dogs with SRMA were younger than dogs with IMPA and therefore likely had not reached a neutering age yet.

In humans, some inflammatory and infectious diseases cause extreme increases in CRP values (>100 mg/L) more frequently than others (10). Infectious diseases are associated with higher CRP than non-infectious conditions, and bacterial infections are accompanied by higher CRP concentrations compared to non-bacterial infections (10). The highest CRP values due to infections are recorded in patients with pneumonia, and the highest CRP values in non-infectious inflammatory diseases are recorded in patients with inflammatory bowel disease. Very high CRP (>200 mg/L) has been found to be a marker of sepsis, while very low CRP (<10 mg/L) was characteristic of cardiovascular diseases and viral infections. Furthermore, a higher CRP value is associated with higher mortality, the need for intubation and longer hospitalization in various human diseases (10, 34).

We found a significant positive correlation between body temperature and CRP concentrations throughout the whole study population, as well as in each final diagnosis. Such correlation has been previously reported in dogs with various diseases (35). A correlation between the CRP and temperature has been described in people, which was additionally influenced by the age of the person. The positive correlation was present up to the cut off age of 40. Additionally, the body temperature in the middle-aged and the elderly patients was lower than in young people with the same CRP concentration (10). Such relationship has never been explored in dogs. Whether it is temperature or CRP which normalizes sooner or which of the two, when abnormal, has a higher diagnostic accuracy for diagnostic sampling remains unknown.

This study has several limitations. It was a retrospective study, in which patients were referred to either neurology or internal medicine departments, and therefore could have led to different diagnostic approaches. Both CSF and synovial fluid should ideally have been collected from all dogs in the study. Unfortunately, only one of these procedures was usually performed based on the most likely diagnosis. Therefore, a number of patients diagnosed with either SRMA or IMPA may have been suffering from both conditions concomitantly (1). The CRP concentration was measured using different assays in both hospitals, however they were both standardized procedures, for which very high level of agreement was shown (26). Another limitation noted is that CRP was measured semi-quantitively (e.g., >100 mg/L, >200 mg/L) in one sixth of the patients, and were replaced with arbitral values which could subsequently reduce precision of our analyses.

In conclusion, dogs diagnosed with SRMA had significantly higher CRP concentration than dogs diagnosed with IMPA. This simple difference was significantly modified by the patient's age. In young dogs (<12 months), a higher CRP value indicated IMPA, whereas in older dogs, higher CRP value indicated SRMA. CRP concentrations should not be used as a sole diagnostic modality to differentiate between SRMA and IMPA, given it only has fair discriminatory potential. The reason for such CRP variability, depending on age and diagnosis, is not known, but it indicates that the individual characteristics of the patient should be taken into account when interpreting results of CRP assays. Further studies to investigate this finding may help to improve our knowledge and understanding of disease pathophysiology in both conditions.

## Data availability statement

The raw data supporting the conclusions of this article will be made available by the authors, without undue reservation.

## Ethics statement

The animal study was reviewed and approved by Research Ethics Committee University of Glasgow. Written informed consent for participation was not obtained from the owners because the data was collected retrospectively.

## Author contributions

VI and JB: hypothesis generation and design of the study, acquisition, analysis and interpretation of data, drafting, and revising the article for intellectual content. SK and RG-Q: design of the study and writing and revising the manuscript. MC: interpreting and analyzing the results and writing and revising the manuscript. All authors approved the submitted version.
