# Supplementary material for: Signalment and C-reactive protein values in dogs with immune-mediated polyarthritis and steroid responsive meningitis arteritis
Source: Front Vet Sci. 2023 Feb 14;10:1091318. doi: 10.3389/fvets.2023.1091318 (PMC9971500; doi:10.3389/fvets.2023.1091318)
Supplement: Supplementary file 1 [file Table_1.docx]

Table S1. Breeds of dogs enrolled in the study – only 149 pedigree dogs are presented

| Breed | SRMA (n=73) | IMPA (n=76) | Total |
| --- | --- | --- | --- |
| Cockapoo | 12 (16.4) | 3 (3.9) | 15 (10.1) |
| Cocker spaniel | 3 (4.1) | 8 (10.5) | 11 (7.4) |
| Whippet | 9 (12.3) | 2 (2.6) | 11 (7.4) |
| Beagle | 9 (12.3) | 1 (1.3) | 10 (6.7) |
| Springer spaniel | 2 (2.7) | 7 (9.2) | 9 (6.0) |
| Sprocker spaniel | 5 (6.8) | 2 (2.6) | 7 (4.7) |
| Weimaraner | 1 (1.4) | 5 (6.6) | 6 (4) |
| Labrador retriever | 2 (2.7) | 3 (3.9) | 5 (3.4) |
| Boxer | 4 (5.5) | 0 (0) | 4 (2.7) |
| Border collie | 3 (4.1) | 1 (1.3) | 4 (2.7) |
| Hungarian Vizsla | 3 (4.1) | 1 (1.3) | 4 (2.7) |
| German shepherd | 1 (1.4) | 3 (3.9) | 4 (2.7) |
| Jack Russel Terrier | 2 (2.7) | 2 (2.6) | 4 (2.7) |
| Shih-tzu | 1 (1.4) | 2 (2.6) | 3 (2.0) |
| Golden retriever | 0 (0) | 2 (2.6) | 2 (1.3) |
| Welsh corgi | 2 (2.7) | 0 (0) | 2 (1.3) |
| Lurcher | 2 (2.7) | 0 (0) | 2 (1.3) |
| Cavapoo | 1 (1.4) | 1 (1.3) | 2 (1.3) |
| Miniature Schnauzer | 1 (1.4) | 1 (1.3) | 2 (1.3) |
| Pointer | 1 (1.4) | 1 (1.3) | 2 (1.3) |
| Poodle | 0 (0) | 2 (2.6) | 2 (1.3) |
| Rhodesian Ridgeback | 0 (0) | 2 (2.6) | 2 (1.3) |
| Bearded collie | 0 (0) | 2 (2.6) | 2 (1.3) |
| English springer spaniel | 0 (0) | 2 (2.6) | 2 (1.3) |
| Cavalier King Charles Spaniel | 0 (0) | 2 (2.6) | 2 (1.3) |
| Rottweiler | 0 (0) | 2 (2.6) | 2 (1.3) |
| Italian greyhound | 1 (1.4) | 0 (0) | 1 (0.7) |
| Border terrier | 1 (1.4) | 0 (0) | 1 (0.7) |
| Grand Basset Griffin | 1 (1.4) | 0 (0) | 1 (0.7) |
| Irish terrier | 1 (1.4) | 0 (0) | 1 (0.7) |
| Bernese mountain dog | 1 (1.4) | 0 (0) | 1 (0.7) |
| Afghan hound | 1 (1.4) | 0 (0) | 1 (0.7) |
| Mini labradoodle | 1 (1.4) | 0 (0) | 1 (0.7) |
| Bichon Frise | 1 (1.4) | 0 (0) | 1 (0.7) |
| Sproodle | 1 (1.4) | 0 (0) | 1 (0.7) |
| Dachshund | 0 (0) | 1 (1.3) | 1 (0.7) |
| Maltese | 0 (0) | 1 (1.3) | 1 (0.7) |
| English bulldog | 0 (0) | 1 (1.3) | 1 (0.7) |
| Flat coat retriever | 0 (0) | 1 (1.3) | 1 (0.7) |
| French bulldog | 0 (0) | 1 (1.3) | 1 (0.7) |
| Toy Poodle | 0 (0) | 1 (1.3) | 1 (0.7) |
| Doberman | 0 (0) | 1 (1.3) | 1 (0.7) |
| Irish wolfhound | 0 (0) | 1 (1.3) | 1 (0.7) |
| Pomeranian | 0 (0) | 1 (1.3) | 1 (0.7) |
| Welsh springer spaniel | 0 (0) | 1 (1.3) | 1 (0.7) |
| Alaskan malamute | 0 (0) | 1 (1.3) | 1 (0.7) |
| Chihuahua | 0 (0) | 1 (1.3) | 1 (0.7) |
| Italian Spinone | 0 (0) | 1 (1.3) | 1 (0.7) |
| Schnauzer | 0 (0) | 1 (1.3) | 1 (0.7) |
| Airedale Terrier | 0 (0) | 1 (1.3) | 1 (0.7) |
| Bedlington Terrier | 0 (0) | 1 (1.3) | 1 (0.7) |
| German shorthaired pointer | 0 (0) | 1 (1.3) | 1 (0.7) |
| Cavachon | 0 (0) | 1 (1.3) | 1 (0.7) |
| English setter | 0 (0) | 1 (1.3) | 1 (0.7) |
